# Seize the engine: Emerging cell cycle targets in breast cancer

**DOI:** 10.1002/ctm2.1544

**Published:** 2024-01-24

**Authors:** Jesús Fuentes‐Antrás, Philippe L. Bedard, David W. Cescon

**Affiliations:** ^1^ Division of Medical Oncology and Hematology Department of Medicine Princess Margaret Cancer Centre University Health Network University of Toronto Toronto Ontario Canada; ^2^ NEXT Oncology Hospital Universitario QuironSalud Madrid Madrid Spain

**Keywords:** breast cancer, cell cycle checkpoints, clinical trials, cyclin‐dependent kinases, drug development, mitotic kinases

## Abstract

Breast cancer arises from a series of molecular alterations that disrupt cell cycle checkpoints, leading to aberrant cell proliferation and genomic instability. Targeted pharmacological inhibition of cell cycle regulators has long been considered a promising anti‐cancer strategy. Initial attempts to drug critical cell cycle drivers were hampered by poor selectivity, modest efficacy and haematological toxicity. Advances in our understanding of the molecular basis of cell cycle disruption and the mechanisms of resistance to CDK4/6 inhibitors have reignited interest in blocking specific components of the cell cycle machinery, such as CDK2, CDK4, CDK7, PLK4, WEE1, PKMYT1, AURKA and TTK. These targets play critical roles in regulating quiescence, DNA replication and chromosome segregation. Extensive preclinical data support their potential to overcome CDK4/6 inhibitor resistance, induce synthetic lethality or sensitise tumours to immune checkpoint inhibitors. This review provides a biological and drug development perspective on emerging cell cycle targets and novel inhibitors, many of which exhibit favourable safety profiles and promising activity in clinical trials.

## Graphical abstract

Cell cycle checkpoint disruption drives breast cancer progression. CDK4/6 inhibitors are a mainstay of treatment for hormone receptor‐positive breast cancer patients. Cell cycle regulators with promising clinical potential include CDK2, CDK4, CDK7, PLK4, WEE1, PKMYT1, AURKA and TTK. Novel inhibitors of these targets, alone or in combination, may overcome CDK4/6 inhibitor resistance, induce synthetic lethality and sensitise tumours to immunotherapy.

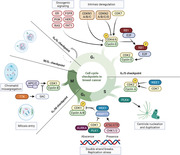

## 1 CELL CYCLE DEPENDENCY AS A THERAPEUTIC VULNERABILITY IN BREAST CANCER

**FIGURE 1:**
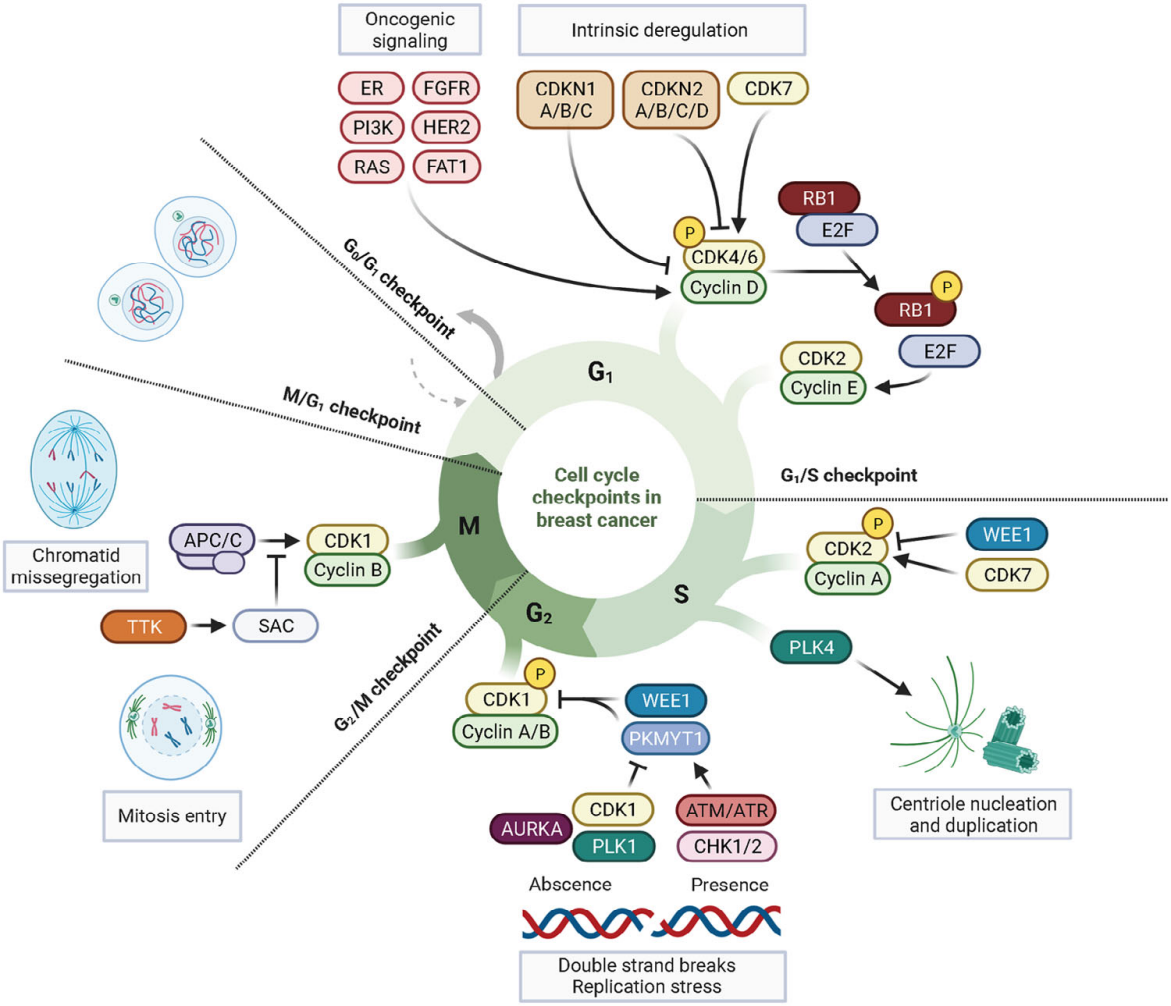
Graphical simplification of cell cycle phases, checkpoints and regulatory mechanisms in breast cancer.

Abnormal activity of the cell cycle and its resulting genomic instability are hallmarks of cancer. Cancer cells are able to override the checkpoints that govern cell cycle progression to achieve unrestrained proliferation despite the accumulation of genomic aberrations. At least four cell cycle checkpoints may be deregulated in breast cancer (BC) cells: the restriction point (G0/G1), the G1 checkpoint (G1/S), the G2 checkpoint (G2/M) and the mitosis‐associated spindle assembly checkpoint (SAC) (Figure 1).^1^ Hormone receptor‐positive, HER2‐negative (HR^+^/HER2^−^) tumours largely rely on oestrogen receptor (ER) signalling and downstream cyclin D1 (*CCND1*) to drive cell cycle progression through the G1/S checkpoint.^2^ Luminal tumours also frequently harbour activating mutations in the PI3K signalling pathway and *CCND1* amplifications.^3^, ^4^ Triple negative (TNBC) and HER2‐positive (HER2^+^) tumours, conversely, more commonly harbour *RB1* loss‐of‐function alterations, altered DNA damage response (DDR), near‐universal loss of *TP53* function, *CCNE1* and *CDK4* amplifications and *PTEN* loss‐of‐function mutations.^5^, ^6^ These alterations allow tumour cells to overcome not only the G1/S but also the G2/M mitotic checkpoints. TNBC tumours also display high levels of genomic instability and aneuploidy secondary to the abrogation of DNA repair mechanisms and deficient function of the SAC. The knowledge of these molecular features, along with the clinical benefit observed with CDK4/6 inhibitors (CDK4/6i) in HR^+^/HER2^−^–BC, provide a rationale for targeting cell cycle regulators in patients with relapsed or metastatic disease that are generally considered incurable and for which additional effective therapies are needed. These approaches are based on diverse mechanistic insights including forcing cancer cells to permanently exit the cell cycle or, conversely, to override checkpoints, impairing replication stress tolerance or inducing catastrophic genomic instability. Here, we review the key drug development efforts to target the cell cycle machinery with a focus on the lessons learned and potential applications in BC.

## 2 G1/S PHASE TRANSITION

### 2.1 CDK4/6

CDK–cyclin complexes are universal drivers of cell cycle transitions. CDK4/6i are the only approved drugs directly targeting the cell cycle in BC, where they force cancer cells to exit the cell cycle into quiescence. Their development has been focused on HR^+^ BC, where underlying genomic (e.g. *CCND1* amplification, *CDK4* amplification, *CDKN2A* loss, intact *RB1*) and transcriptomic (ER‐ or PI3K‐dependent *CCND1* overexpression and activity) features render higher sensitivity to the inhibition of the cyclinD‐CDK4/6 pathway.^7^, ^8^, ^9^, ^10^, ^11^, ^12^ In particular, palbociclib, ribociclib and abemaciclib are third‐generation selective CDK4/6i whose use is well established in HR^+^/HER2^−^ metastatic BC (mBC) (Table S1).^13^, ^14^, ^15^, ^16^, ^17^, ^18^, ^19^, ^20^, ^21^, ^22^, ^23^, ^24^ These drugs are United States Food and Drug Administration (US FDA) and European Medicines Agency approved for the treatment of HR^+^/HER2^−^–mBC in combination with endocrine therapy (ET), while abemaciclib is also approved as monotherapy after progression to ET and chemotherapy. Dalpiciclib, which has been studied in China, also led to survival improvements in combination with fulvestrant in endocrine‐resistant disease.^25^, ^26^

In the early BC setting, abemaciclib was recently approved in combination with ET for the adjuvant treatment of high risk early HR^+^/HER2^−^ BC following the results of the MonarchE trial, and initial reports of the adjuvant NATALEE ribociclib trial are positive.^27^, ^28^ In contrast, the adjuvant palbociclib trial failed to meet its primary endpoint of improving invasive disease‐free survival (IDFS).

A variety of trials have explored the impact of adding CDK4/6i to neoadjuvant ET on the pathological complete response rate as well as on other surrogate endpoints (e.g. pre‐operative endocrine prognostic index score, Ki67 index, PAM50 risk of relapse score).^29^, ^30^, ^31^, ^32^, ^33^, ^34^, ^35^ While these neoadjuvant trial results are encouraging, the lack of event‐free survival data and the heterogeneity in trial design, particularly regarding the use of different early biological endpoints, preclude the comparison among agents and translation to clinical practice.

In sum, while all CDK4/6i prolong progression‐free survival (PFS), palbociclib has not shown benefits in overall survival (OS) and IDFS. Whether this reflects true differences between these agents, or rather in the trial designs, is unclear.^36^, ^37^ In this regard, palbociclib exhibits comparable potency against cyclin D1/CDK4 and cyclin D2/CDK6, whereas ribociclib and abemaciclib display greater potency against CDK4, with abemaciclib also blocking CDK1, CDK2, CDK5 and CDK9. Additionally, the continuous dosing of abemaciclib differs from the 21 days on, 7 days off regimen of its counterparts, potentially influencing the development of biologically distinct resistant populations. Comprehensive reviews addressing the differences between CDK4/6i are available elsewhere.^38^, ^39^, ^40^

Other CDK4/6i, such as lerociclib or trilaciclib, remain largely investigational at present. Lerociclib, a continuous oral CDK4/6i, has shown a safety profile and preliminary efficacy comparable to the approved agents in HR^+^/HER2^−^–mBC.^41^ The development of trilaciclib, which aims to maintain immune and bone marrow cells in G1 arrest and protect them from chemotherapy‐induced damage, has mostly focused on TNBC, where it has been granted an US FDA fast track designation, and is currently being evaluated in the phase 3 PRESERVE 2 study.^42^, ^43^

Intrinsic resistance to CDK4/6i is uncommon but acquired resistance to therapy eventually emerges in most patients. Identifying effective therapies following progression on CDK4/6i in HR^+^/HER2^−^–BC patients is an area of urgent clinical need considering the limited activity of single‐agent fulvestrant following progression.^44^, ^45^ While acquired CDK4/6i cross‐resistance is frequent in BC models, there is preclinical evidence supporting the use of abemaciclib after progression on palbociclib.^46^ A retrospective, multi‐centre cohort of 87 patients treated with abemaciclib after progression to palbociclib reported a 6‐month PFS of 37%, and a randomised phase 3 trial of this strategy is ongoing.^47^ Consistently, the MAINTAIN phase II trial reported that the combination of ET plus ribociclib showed a modest benefit over fulvestrant monotherapy after progression on first‐line ET plus CDK4/6i (84% of patients had received palbociclib), adding to the potential role for CDK4/6i switching following progression.^48^ In contrast, the PACE study, evaluating palbociclib added to fulvestrant after progression on an AI plus CDK4/6i (mostly palbociclib), failed to show benefit.^49^ The question of whether the utility of continuation of a CDK4/6i‐based strategy following initial progression depends on the biology of resistant disease is just beginning to be explored.

### 2.2 Mechanisms of resistance to CDK4/6i

As more patients have been treated with CDK4/6i, correlative analyses have shed light on potential resistance mechanisms. From a mechanistic standpoint, genomic alterations associated with resistance may be divided into two categories: resistance through the disruption of the cell cycle machinery (including alterations in *RB1, CCNE1, CDK6, CDK4, p16, CDK2, CDK7, CCND1* or *INK*) and resistance through compensatory upstream signalling (alterations in PI3K, PTEN, FGFR1/2, FAT1/HIPPO, ERBB2 or RAS signalling) (Figure 1).^50^

Most of the efforts regarding targeted therapy to date have focused on inhibiting the PI3K/AKT/mTOR pathway, based on work that was underway before the widespread introduction of CDK4/6i use in the first line. As activating mutations in PI3K tend to be truncal rather than acquired alterations, and thus are not specifically acquired with CDK4/6i resistance, the activity of these agents following progression on CDK4/6i could be influenced by ongoing dependence on this oncogenic signalling in resistant disease. With the initial evaluation of the PI3Kα‐selective inhibitor alpelisib conducted in patients mostly untreated with CDK4/6i, the phase 2 BYLieve trial was carried out to evaluate alpelisib in CDK4/6i pre‐treated participants. Notably, this trial reported a 6‐month PFS of nearly 50%.^51^ The confirmatory phase 3 randomised EPIKB5 trial was recently launched. A phase 1/2 trial evaluating inavolisib, an investigational PI3Kα‐selective inhibitor, in combination with fulvestrant, recently showed a favourable safety profile compared to other agents of the same class and an encouraging preliminary anti‐tumour activity with an overall response rate (ORR) of 25% and a clinical benefit rate (CBR, defined as the sum of complete response, partial response and stable disease > 6 months) of 49% in a similar population of patients with *PIK3CA*‐mutated HR^+^/HER2^−^–BC following progression on ET and CDK4/6i.^52^ Alpelisib and inavolisib will be compared in this setting, in combination with fulvestrant, in the randomised phase 3 INAVO121 study.^53^ Data from ongoing studies testing mutant‐selective PI3K inhibitors LOXO783 or RLY‐2608 that may provide a more favourable therapeutic index are also eagerly awaited.

Within the same pathway, the phase 3 CAPItello‐291 trial recently reported that the addition of the AKT inhibitor capivasertib to fulvestrant improved PFS in endocrine‐resistant patients, including 60% who had progressed on a CDK4/6i.^54^ The ongoing phase 3 FINER study testing ipatasertib will provide further data about the benefit of an AKT inhibitor in the immediate post‐CDK4/6i setting. Additionally, there is encouraging retrospective data supporting the similar benefit in survival outcomes of exemestane plus mTOR inhibitor everolimus irrespective of prior CDK4/6i exposure, and the benefit of the triplet exemestane, everolimus and ribociclib beyond CDK4/6i progression is currently being investigated in the phase 1/2 TRINITI‐1 trial.^55^, ^56^

The pan‐HER2 inhibitor neratinib and FGFR1‐4 kinase inhibitor rogaratinib for patients with *ERBB2* mutations or *FGFR1*/2/3 amplification, respectively, are other examples of targeting compensatory upstream signalling in the post‐CDK4/6i setting, and combinations with other agents, including CDK4/6i, are being developed.^57^, ^58^

### 2.3 CDKs beyond CDK4/6

**FIGURE 2:**
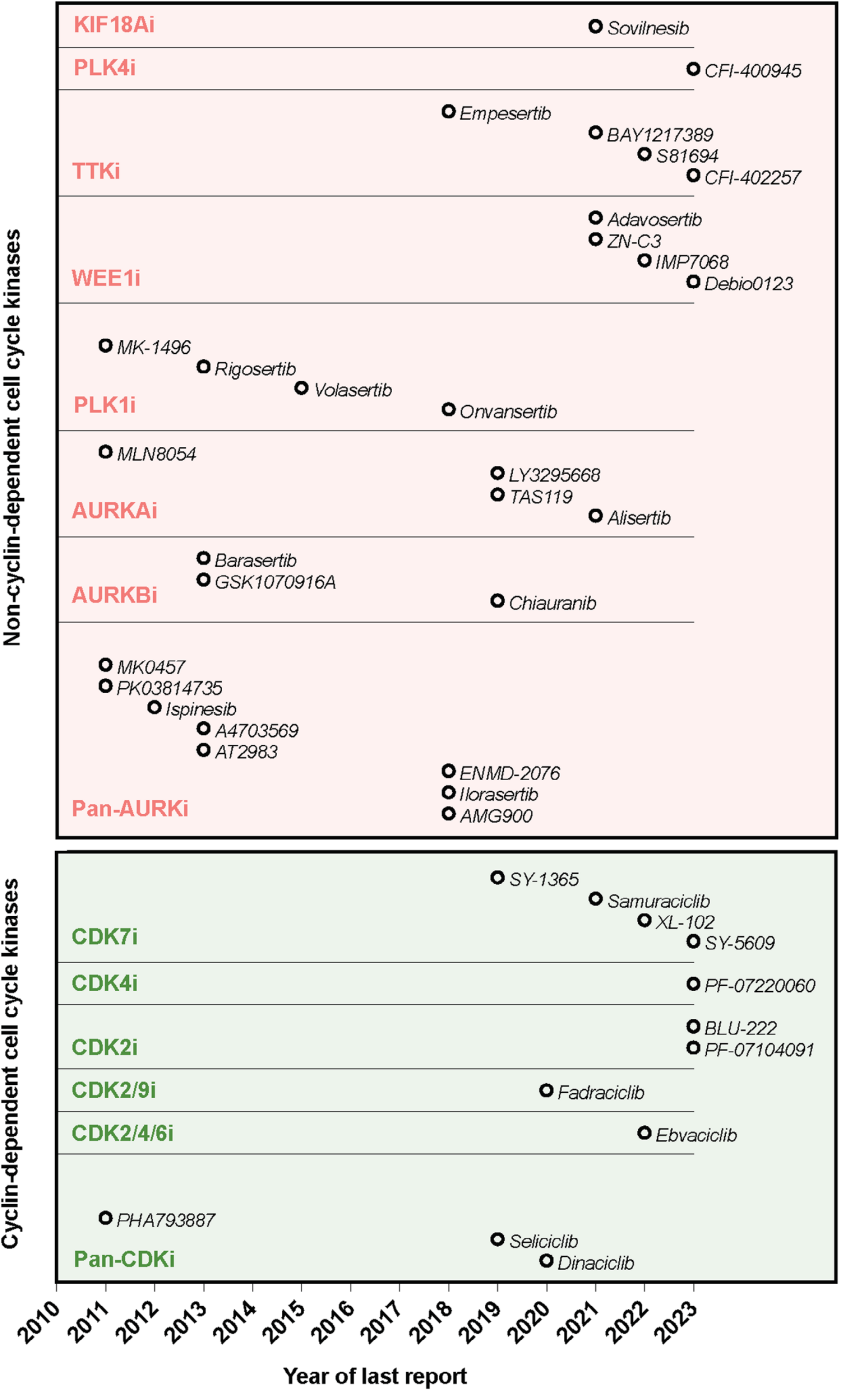
Temporal overview of drug development targeting the cell cycle. The year of the last report of clinical data since 2010, either journal article or conference abstract, is shown. Further detail is provided in Tables 1 and 2, including drugs with no available clinical data at present.

The components of the CDK family can be functionally classified into those directly intervening in the cell cycle process (CDK1, CDK4, CDK5, CDK6) and those acting as transcriptional regulators (CDK7, CDK8, CDK9, CDK11, CDK19, CDK20; of which only CDK7, CDK11 and CDK20 act as regulators of cell cycle progression).^59^ A number of pan‐CDK inhibitors (CDKi) have been studied over the last two decades with generally limited anti‐tumour activity and considerable toxicity (Figure 2 and Table 1). The first non‐selective CDKi to enter clinical testing, alvocidib, was shown to produce potent cell cycle arrest and apoptosis in preclinical models.^60^ However, alvocidib showed minimal clinical anti‐tumour efficacy as a single agent in solid tumours, while combination trials with docetaxel or cisplatin produced intolerable neutropenia or gastrointestinal side effects.^61^, ^62^ Other non‐selective inhibitors such as R547, riviciclib, PHA‐793887 and seliciclib also failed to advance in clinical development due to limited activity and/or poor tolerability.^63^, ^64^, ^65^, ^66^ Dinaciclib, a CDK 1, 2, 5 and 9 inhibitor, reached late stage clinical development but was ultimately terminated due to limited activity in advanced BC either alone or in combination with epirubicin or pembrolizumab (Table 1).^67^, ^68^, ^69^

**TABLE 1:** Drug development targeting cyclin‐dependent kinases (CDKs) beyond CDK4/6. Clinical evidence and ongoing trials are presented, with a special focus on those with breast cancer cohorts.

Cell cycle target	Drug	Stage	NCT	Patient cohort	Status	Population (*n*)	Intervention	Efficacy	Safety	References (if applicable)	Year of publication
CDK1/2/4/6/7/9	Flavopiridol (Alvocidib)	1/2	NCT00020332	mBC	Completed	11	Alvocidib + docetaxel	ORR 9%, DCR 18%	DLT 45%	^61^	2004
		1	NCT00039455	mBC	Terminated		Alvocidib + trastuzumab				
		1	NCT00003690	Various	Completed	39	Alvocidib + platin	ORR 0%, CBR 34%	G≥3 nausea (30%), diarrhoea (15%), neutropenia (10%)	^62^	2005
CDK 1/2/4/7/9	R547	1	NCT00400296	Various	Completed	41	R547	ORR 6%	Low grade and reversible. G≥3 individual cases of fatigue, nausea, pruritus, somnolence	^63^	2007
CDK1/4/9	Riviciclib (P276‐00)	1	NCT01333137	mTNBC	Terminated		Carboplatin + gemcitabine + Alvocidib				
CDK1/2/4	PHA‐793887	1	NCT00996255	Various	Terminated (liver toxicity)	19		ORR 0%, DCR 26%	DLT liver toxicity 26% including a fatal hepatorenal failure	^64^	2011
CDK1/2/5/7/9	Seliciclib		NCT00999401	Various mBC cohort	Completed	mBC *BRCA1/2m* (*n* = 20)	Seliciclib + sapacitabine	ORR 10%, CBR 30%	G≥3 neutropenia 25%, LFT increase 20%	^65^, ^66^	2016, 2019
			NCT01333423	mTNBC	Withdrawn		Seliciclib + liposomal doxorubicin				
CDK1/2/5/9	Dinaciclib (SCH 727965, MK7965)	1/2	NCT00732810	Various mBC and NSCLC	Completed	mBC (*n* = 13)	Dinaciclib vs. capecitabine	ORR 8%	G≥3 74%, G≥3 neutropenia 47%	^67^	2014
			NCT01624441	mTNBC	Completed	9	Dinaciclib + epirubicin	ORR 0%	G≥3 febrile neutropenia 22%, syncope 22%, nausea 11%	^68^	2015
			NCT01676753	mTNBC	Completed	46	Dinaciclib + pembrolizumab	ORR 17%, DCR 38%	G≥3 neutropenia 38%	^69^	2020
CDK2	PF‐07104091	1/2	NCT04553133	Various HR^+^HER2^−^ pre/post CDK4/6i	Recruiting	mBC (*n* = 16)	PF‐07104091 (combos with ET, CDK4/6i)	ORR 19%, DCR 62%	G≥3 57% (nausea 15%, diarrhoea 9%, fatigue 20%)	^80^	2023
		1/2	NCT05262400	Various HR^+^HER2^−^	Recruiting		PF‐07104091 + CDK4i + ET				
	BLU‐222	1/2	NCT05252416	Various	Recruiting		BLU‐222 ± fulvestrant + ribociclib or carboplatin		All grade nausea 26%, diarrhoea 22%, anaemia 19%	^81^	2023
	ARTS‐021	1	NCT05867251	Various HR^+^HER2^−^	Not yet recruiting						
CDK4	PF‐07220060	1/2	NCT05262400	Various HR^+^HER2^−^ post CDK4/6i	Recruiting	HR^+^HER2^−^ post CDK4/6i (*n* = 21)	PF‐07220060 + CDK2i + ET	ORR 29%, CBR 52%	All‐grade diarrhoea 50%; (0% G≥3), neutropenia 50% (15% G≥3) and nausea 39%; 3.8% G≥3)	^82^	2023
CDK2/4/6	NUV‐422	1/2	NCT04541225	Various	Terminated (uveítis)		NUV‐422				
		1/2	NCT05191004	HR^+^HER2−pre/post CDK4/6i	Withdrawn		NUV‐422 + fulvestrant				
	Ebvaciclib (PF‐06873600)	1/2	NCT03519178	HR^+^HER2^−^ pre/post CDK4/6i, mTNBC, ovary	Active, not recruiting	HR^+^HER2^−^ (*n* = 59), TNBC (*n* = 2), ovary (*n* = 6)	Ebvaciclib ± fulvestrant	DCR 48% (E)−67% (E+F)	DLT 13% G≥3 neutropenia 16%, anaemia 14%	^78^	2022
CDK2/9	Fadraciclib (CYC065)	1/2	NCT02552953	Various	Active, not recruiting	24	Fadraciclib	ORR 4%	–	^235^	2020
		1/2	NCT04983810	Various. HR^+^HER2^−^ post CDK4/6i, mHER2^+^, mTNBC	Recruiting						
CDK7	SY‐5609	1	NCT04247126	Various	Recruiting	HR^+^HER2^−^ post CDK4/6i (*n* = 12)	SY‐5609 ± fulvestrant	ORR 0%, CBR 25%	G≤2 nausea 36%, diarrhoea 29%	^84^, ^85^	2021, 2023
	SY‐1365	1	NCT03134638	Various	Terminated (business decision)	80	SY‐1365 ± carboplatin	DCR 44% (of 18 pts in expansion)	Low grade and reversible	^83^	2019
	LY3405105	1	NCT03770494	Various	Terminated (lack of efficacy)	50	LY3405105	ORR 0%, DCR 34%	All‐grade diarrhoea (33%), nausea (19%), fatigue (15%)	^88^	2023
	XL102	1	NCT04726332	Various	Recruiting	mBC (*n* = 12)	XL102		All‐grade nausea (38%), diarrhoea (42%), G≥3 0%. G≥3 anaemia 12%	^89^	2022
	Samuraciclib (CT7001)	1/2	NCT03363893	Various	Active, not recruiting	44	Samuraciclib	DCR 53% (64% at RP2D)	G≥3 21%. G≤2 diarrhoea, nausea, vomiting 77%	^236^, ^237^	2021
		1/2	NCT03363893	HR^+^HER2^−^ post CDK4/6i	Active, not recruiting	31	Samuraciclib + fulvestrant	ORR 8%, CBR 39%	G≥3 42% of which 19% diarrhoea. 90% all grade diarrhoea	^86^	2021
		1/2	NCT04802759	HR^+^HER2^−^post CDK4/6i, mTNBC	Recruiting		Samuraciclib + giredestrant				
CDK8	BCD‐115	1	NCT03065010	HR^+^HER2^−^pre/post CDK4/6i	Completed						
CDK9	PRT2527	1	NCT05159518	Various (mTNBC, HR^+^HER2^−^ post CDK4/6i)	Recruiting						
	KB‐0742	1	NCT04718675	Various	Recruiting						

Abbreviations: CBR, clinical benefit rate; CDK4/6i, CDK4/6 inhibitor; DCR, disease control rate; DLT, dose limiting toxicity; HR^+^/HER2^−^, hormone receptor‐positive, HER2‐negative; mBC, metastatic breast cancer; ORR, objective response rate; TNBC, triple negative breast cancer.

The recent success of CDK4/6i and the improved understanding of genomic vulnerabilities spurred a new wave of development of selective CDKi in solid tumours. An increased expression and activity of CDK2 and CDK7 has been found in CDK4/6i‐resistant cell models of HR^+^/HER2^−^ and TNBC.^70^, ^71^, ^72^ While the cyclin E‐CDK2 complex participates in the G1/S phase transition through RB1 phosphorylation, CDK7 exerts a wider role in cell cycle progress by acting as a CDK‐activating kinase (Figure 1).^73^ In vitro inhibition of CDK2 or CDK7 has been shown to restore endocrine sensitivity and overcome CDK4/6i resistance.^74^, ^75^, ^76^, ^77^ Clinical trials with compounds selectively blocking CDK2 or CDK4 are ongoing, some of them as combinations with ET and CDK4/6i in HR^+^/HER2^−^–mBC. In the dose escalation part of the phase 1/2a trial testing the first‐in‐class CDK2/4/6i PF‐06873600 (ebvaciclib), an acceptable safety profile was observed along with preliminary evidence of anti‐tumour activity in 59 HR^+^/HER2^−^–BC patients who had progressed on a previous CDK4/6i and ≤2 prior chemotherapy lines.^78^ This included the achievement of a disease control rate (DCR, indicating the achievement of complete response, partial response or stable disease as best response) of 48% (28 out of 58) in the monotherapy arm and 67% (six out of nine) in combination with fulvestrant.^78^ However, ebvaciclib is no longer advancing in BC, and the development of another CDK2/4/6i, NUV‐422, was terminated early due to the unexpected observation of uveitis during dose escalation.^79^

More selective agents targeting CDK2 or CDK4 have recently produced promising results (Table 1). For instance, the selected CDK2i PF‐07104091 elicited a 19% ORR and 62% DCR among 16 heavily pre‐treated post‐CDK4/6i HR^+^/HER2^−^–mBC patients; however, there were notable toxicities including 77% all‐grade nausea (14% ≥G3), 50% diarrhoea (9% ≥G3) and 46% fatigue (20% ≥G3).^80^ In a similar setting, CDK2i BLU‐222 is undergoing dose escalation and has shown a partial response in one out of 12 BC patients, a case of HR^+^/HER2^−^–mBC that had received palbociclib, abemaciclib and capecitabine.^81^ More favourably, among 21 evaluable patients treated with the selective CDK4i PF‐07220060, ORR was 29% and CBR was 54%, with better tolerability including 50% all grade diarrhoea (0% ≥G3), 50% neutropenia (15% ≥G3) and 39% nausea (4% ≥G3).^82^

The first‐in‐class selective CDK7 inhibitor, intravenous SY‐1365, exhibited frequent gastrointestinal toxicity and limited activity and its development was terminated to prioritise oral SY‐5609.^83^, ^84^, ^85^ SY‐5609 has been safe and tolerable in a variety of dosing schedules, and its combination with fulvestrant has so far resulted in a CBR of 25%, with no responses, among 12 evaluable patients with heavily pre‐treated, post‐CDK4/6i HR^+^/HER2^−^–mBC.^85^ The molecule most advanced in clinical testing is CT7001 (samuraciclib), which recently reported safety and preliminary efficacy data from a modular phase 1/2a trial including post‐CDK4/6i HR^+^/HER2^−^–mBC and TNBC patients. Samuraciclib's adverse event profile included frequent, low‐grade gastrointestinal toxicities such as diarrhoea (90%), nausea (81%) and vomiting (52%). In combination with fulvestrant, a 24‐week CBR of 39%, including two partial responses (8%), was reported among 24 evaluable HR^+^/HER2^−^–mBC patients.^86^ In the TNBC cohort, composed of 21 evaluable women who had received prior taxane and anthracycline, samuraciclib achieved a 24‐week CBR of 24%, including one (5%) partial response.^87^ A combination of samuraciclib with the oral selective ER degrader (SERD) giredestrant is currently being tested within the MORPHEUS phase 1/2b umbrella study, and combinations with elacestrant and ER proteolysis targeting chimera ARV‐471 are also planned. Other CDK7i recently in development include LY3405105 and XL‐102, with both showing a lower incidence of all‐grade gastrointestinal toxicity and mild myelotoxicity, but limited anti‐tumour activity including ORR 0% with the former agent.^88^, ^89^ While the improved toxicity profile is encouraging, more data on anti‐tumour activity are awaited.

The inhibition of other CDKs such as CDK8/19 and CDK9 has demonstrated preclinical efficacy in BC, including models of TNBC and of endocrine‐ and CDK4/6i‐resistant HR^+^/HER2^−^–BC, but this strategy has not yet rendered clinical data.^90^, ^91^, ^92^, ^93^ In particular, more than 15% of breast tumours harbour alterations in CDK8/19, mostly copy number gains, and its expression is inversely correlated with ER expression and relapse‐free survival.^91^

## 3 S PHASE

Entry into S phase is essentially controlled by the CDK2/cyclinA complex, where CDK2 is activated by CDK4/6‐dependent E2F upregulation and by CDK7, and inhibited by DNA damage‐mediated cdc25A and WEE1 (Figure 1). The DNA‐damage response pathway is thus critical for cell cycle regulation in the S but also G2‐M checkpoints. In particular, DNA double‐strand breaks activate the ATM–CHK2–cdc25A axis that prevents S phase entry, while single‐strand DNA damage induces the ATR‐CHK1‐cdc25A axis that limits G2/M progression, with substantial overlapping of the two pathways including WEE1 activation that blocks CDK2 (S phase checkpoint) and CDK1 (G2/M checkpoint). These proximal DNA damage‐induced regulatory pathways and their various effects on the cell cycle have been reviewed elsewhere and fall beyond the scope of our review, where we aimed to focus on more distal kinases amenable to drug development.^94^, ^95^

A key milestone of the S phase is centrosome duplication. Centrosomes are the microtubule organising centres in eukaryotic cells and are composed of two barrel‐shaped organelles embedded in a matrix of proteins known as pericentriolar material. Their duplication in the S phase is critical for the equal distribution of genetic material in mitosis. This process is governed by highly conserved molecular mechanisms that prevent the generation of multipolar spindles and subsequent aneuploidy and genomic instability. In humans, centriole initiation requires PLK4 serine/threonine kinase activity (Figure 1).^96^ Although the specific underlying mechanisms remain incompletely understood, PLK4 provides a mitotic checkpoint to sense DNA damage and other cellular abnormalities.

The identification of PLK4 as a promising therapeutic target resulted from a systematic approach combining kinome‐wide RNAi screening and gene expression analysis in cell lines and human BC.^97^ PLK4 has been found overexpressed in BC of all subtypes and its increased activity has been consistently linked to disease aggressiveness and epithelial–mesenchymal transition in vitro and in vivo.^97^, ^98^, ^99^, ^100^, ^101^ This is likely explained by centrosome amplification, multipolarity and resulting aneuploidy and genomic instability. PLK4 upregulation is associated with a higher incidence of lymph node metastasis, distant metastasis, shorter survival and worse response to neoadjuvant taxane‐based chemotherapy and adjuvant tamoxifen.^98^, ^102^, ^103^ ^,^ ^104^

Several small‐molecule PLK4 inhibitors have been developed with varying selectivity (Figure 2 and Table 2). These include CFI‐400945, centrinone, centrinone B and YLT‐11, all of which interact with the ATP‐binding pocket in the catalytic domain of PLK4. Of these, CFI‐400945, has proceeded to clinical development as a first‐in‐class oral PLK4 inhibitor, identified through an academic discovery program. Anti‐tumour activity of CFI‐400945 was demonstrated in human BC xenografts representing all recognised BC subtypes.^97^ CFI‐400945 blocks autophosphorylation of PLK4, which is a critical step for its activation, and its anti‐tumour effects may depend on facilitating errors in chromosome segregation and genomic instability. Recently, our group demonstrated the synergistic anti‐proliferative effect of CFI‐400945 in combination with radiotherapy in TNBC models.^105^ Centrinone A and B are the most selective PLK4 inhibitors and have been extensively studied as preclinical tool compounds, but lack pharmacologic properties for clinical drug development.^106^, ^107^ Last, YLT‐11, the most recently described small‐molecule inhibitor, has shown high PLK4 selectivity and remarkable anti‐proliferative activity in vitro and in human BC xenografts.^108^

**TABLE 2:** Drug development targeting non‐cyclin‐dependent cell cycle kinases. Clinical evidence and ongoing trials are presented, with a special focus on those with breast cancer cohorts.

Cell cycle target	Drug	Stage	NCT	Patient cohort	Status	Population (*n*)	Intervention	Efficacy	Safety	References (if applicable)	Year of publication
PLK4	CFI‐400945	1	NCT01954316	Various	Completed	52	CFI‐400945	ORR 2%, CBR 8%	G≥3 neutropenia 19%	^109^	2019
		2	NCT04176848	mTNBC	Active, not recruiting	15	CFI‐400945 + durvalumab	ORR 0%, DCR 7%	G≥3 neutropenia 20%	^111^	2023
		2	NCT03624543	mBC	Recruiting	27	CFI‐400945	ORR 11%, CBR 22%	G≥3 neutropenia 64%	^110^	2023
TTK	CFI‐402257	1/2	NCT02792465	Various HR^+^HER2^−^ mBC	Active, not recruiting	HR^+^HER2^−^ mBC (n = 20)	CFI‐402257 (+ fulvestrant in cohort C)	ORR 10%, DCR 25%	G≥3 neutropenia 5%	^194^, ^196^, ^238^, ^239^	2023
		1/2	NCT03568422	HER2^−^ mBC	Active, not recruiting	37	CFI‐402257 + paclitaxel	ORR 8%, CBR 55%	G≥3 neutropenia 70%	^240^	2023
	Empesertib (BAY1161909)	1	NCT02138812	Various	Terminated (strategic decision)	69	BAY1161909 + paclitaxel	ORR 14%, DCR 48%	G≥3 events 16−28%	^190^	2018
	BAY1217389	1	NCT02366949	Various	Completed	75	BAY1217389 + paclitaxel	ORR 32%, CBR 78%	G≥3 neutropenia 32%, febrile neutropenia 16%, anaemia 23%	^191^	2021
	S81694	1/2	NCT03411161	Various	Completed	38	S81694	ORR 3%, CBR 40%	G≥3 29% with neutropenia 11%	^197^	2022
	BOS 172722	1	NCT03328494	Various	Completed		BOS 172722 + paclitaxel				
KIF18A	Sovilnesib (AMG650)	1	NCT04293094	Various	Completed					^202^	2021
	VLS‐1488	1	NCT05902988	Various	Not yet recruiting						
WEE1	Adavosertib (AZD1775)	1/2	NCT02482311	Various	Completed	25	Adavosertib	ORR 2/8 gBRCAm pts	Low‐grade and reversible	^128^	2015
		2	NCT03330847	mTNBC	Active, not recruiting	47	Adavosertib + olaparib	–	Terminated due to frequent G≥3 haematological toxicity	^219^	2015
		2	NCT03012477	mTNBC	Completed	37	Adavosertib + cisplatin	ORR 26%	G≥3 53% including diarrhoea 21%, neutropenia 18%	^132^	2021
	IMP7068	1	NCT04768868	Various	Recruiting	24	IMP7068	CBR 64%	G≥3 13%	^136^	2022
	ZN‐c3	1	NCT04158336	Various	Recruiting	39	ZN‐c3	ORR 13%, CBR 44%	Not specified	^137^	2021
	ZN‐c3	1	NCT05368506	mTNBC, ovarian	Not yet recruiting		ZN‐c3				
	Debio 0123	1	NCT05109975	Various	Recruiting		Debio 0123				
	Debio 0123	1	NCT03968653	Various	Recruiting		Debio 0123 + carboplatin (cycle 2 onwards)	–	G≥3 thrombocytopenia 8%; all grade nausea 32%, anaemia 21%	^140^	2023
PKMYT1	RP‐6306	1	NCT04855656	Various	Recruiting		RP‐6306 + RP‐3500 (ATRi)				
		1	NCT05147272	Various	Recruiting		RP‐6306 + gemcitabine				
		1	NCT05147350	Various	Recruiting		RP‐6306 + FOLFIRI				
		2	NCT05601440	HR^+^HER2^−^ post CDK4/6i	Recruiting		RP‐6306 + gemitabine				
PLK1	Volasertib (BI 6727)	1	NCT00969553	Various	Completed	59	Volasertib	ORR 3%, DCR 44%	G≥3 neutropenia 50%, thrombopenia 47%	^171^	2014
		1	NCT02273388	Various	Completed		Volasertib				
		1	NCT00969761	Various	Completed	61	Volasertib + platin	ORR 7%, DCR 34%	G≥3 neutropenia 48%, thrombocytopenia 33%	^172^	2015
		1	NCT01022853	Various	Completed	30	Volasertib + nintedanib	ORR 3%, DCR 60%	–	^173^	2015
		1	NCT01206816	Various	Completed	57	Volasertib + afatinib	ORR 4%, DCR 32%	G≥3 neutropenia 35%, thrombopenia 23%	^174^	2015
	CYC140	1/2	NCT05358379	Various	Recruiting		CYC140				
	Onvansertib (NMS‐1286937)	1	NCT01014429	Various	Completed	21	Onvansertib	ORR 0%, DCR 31%	G≥3 37%, G≥3 neutropenia 16%	^241^	2018
	TAK‐960	1	NCT01179399	Various	Terminated						
	BI 2536	1	NCT02211872	Various	Completed	42	BI 2536	ORR 3%, DCR 42%	G≥3 neutropenia 45%	^242^	2008
	Rigosertib	1	NCT01538537	Various	Completed	29	Rigosertib	ORR 0, DCR 41%	G≥3 anaemia, neutropenia NR (leukopenia 3%)	^175^	2013
		1	NCT01125891	Various	Completed	40	Rigosertib + gemcitabine	ORR 10%, DCR NR	G≥3 neutropenia 26%	^176^	2012
	MK‐1496	1	NCT00880568	Various	Completed	17	MK‐1496	ORR 12%, DCR NR	G≥3 neutropenia 35%, thrombopenia 29%	^243^	2011
AURKA	Alisertib (MLN8237)	1/2	NCT01045421	Various (mBC cohort)	Completed	53 (mBC cohort)	Alisertib	ORR 18%, DCR, 69%, CBR 38%	G≥3 neutropenia 43%, anaemia 10%	^161^, ^244^	2015
		1	NCT00500903	Various	Completed	87	Alisertib	ORR 1%, DCR 39%	G≥3 38%, neutropenia 30%	^245^	2012
		1	NCT00651664	Various	Completed	59	Alisertib	ORR 2%, DCR 37%	G≥3 42%, neutropenia 32%, thrombocytopenia 22%	^246^	2012
		1	NCT01639911	Various	Completed	27	Alisertib + pazopanib	ORR 7%, DCR 63%	G≥3 32%, neutropenia 22%, hypertension 15%	^247^	2019
		2	NCT02187991	HR^+^HER2^−^ mBC	Completed	139	Paclitaxel 90 vs. paclitaxel 60 + alisertib	PFS HR 0.56 (10.2 vs. 7.1 mo)	G≥3 85 vs. 49%, neutropenia 60 vs. 16%, diarrhoea 11 vs. 0%	^160^	2021
		1	NCT01094288	Various	Completed	35	Alisertib + docetaxel	ORR 55%, DCR 100% (in 11 CRPC patients)	DLT 31%, G≥3 neutropenia 86% febrile neutropenia 23%, stomatitis 14%	^248^	2014
		1	NCT02219789	HR^+^HER2^−^ mBC (endocrine resistant, no prior CDK4/6i)	Completed	10	Alisertib + fulvestrant	1‐year PFS 56%, mPFS 12.4 mo	G≥3 neutropenia 22%	^158^	2018
		2	NCT02860000	HR^+^HER2^−^ mBC (endocrine resistant, prior CDK4/6i)	Active, not recruiting	90	Alisertib +/‐ fulvestrant	ORR 20 vs. 18%, CBR 42 vs. 31%, mPFS 5.6 vs. 5.1 mo	G≥3 neutropenia 42 vs. 42%, anaemia 16 vs. 9%	^159^	2021
		1	NCT01613261	Various	Withdrawn (limited activity TAK‐733)		Alisertib + TAK‐733				
	MLN8054	1	NCT00249301	Various	Terminated	61	MLN8054	ORR 0%, DCR 15%	G≥3 21%, somnolence 18%	^249^	2011
	LY3295668	1	NCT03955939	HR^+^HER2^−^ mBC post CDK4/6i	Completed		LY3295668				
		1	NCT03092934	Various	Completed	12	LY3295668	CBR 17%	Low‐grade and reversible	^250^	2019
	TAS‐119	1	NCT02448589	Various	Terminated	72	TAS‐119	ORR 0%, DCR 38%	DLT 16%, G≥3 > 10% diarrhoea, increased lipase	^251^	2019
		1	NCT02134067	Various	Terminated	32	TAS‐119 + paclitaxel	ORR 14%, DCR 59%	G≥3 not specified	^252^	2019
AURKB	GSK1070916A	1	NCT01118611	Various	Completed	32	GSK1070916A	ORR 3%, DCR 63%	G4 neutropenia 28%	^253^	2013
	Chiauranib	1	NCT02122809	Various	Completed	18	Chiauranib	ORR 0%, DCR 67%	G≥3 neutropenia 11%, hypertension 11%	^254^	2019
		2	NCT05336721	mTNBC	Recruiting		Chiauranib + capecitabine				
	Barasertib (AZD1152)	1	NCT00338182	Various	Completed	35	Barasertib	ORR 0%, DCR 23%	G≥3 neutropenia 34%	^255^	2013
Pan‐AURK (A, B, C +/‐ VEGFR)	AMG 900	1	NCT00858377	Various	Completed	105	AMG 900	ORR 3%, DCR 58%	G≥3 75%, neutropenia 42%, thrombocytopenia 14%	^256^	2018
	Ispinesib	1	NCT00363272	Various	Completed	30	Ispinesib	ORR 0%, DCR 30%	G≥3 17%, neutropenia	^257^	2011
		1	NCT00119171	Various	Completed	24	Ispinesib + carboplatin	ORR 0%	G≥3 not specified	^258^	2006
		1	NCT00169520	Various	Completed	24	Ispinesib + docetaxel	ORR 0%, DCR 29%	G≥3 neutropenia 75%	^259^	2008
		1	NCT00607841	mBC	Terminated	16	Ispinesib	ORR 7%, DCR 60%	G3 neutropenia 38%, G4 44%	^260^	2012
		2	NCT00089973	mBC	Completed						
	CYC116	1	NCT00560716	Various	Terminated (sponsor decision)						
	AT9283	1	NCT00443976	Various	Completed	35	AT9283	ORR 3%, DCR 16%	G≥3 neutropenia 25%, G≤2 fatigue 71%	^261^	2013
	MK‐0457	1	NCT02532868	Various	Terminated	27	MK‐0457	ORR 0%, DCR 44%	G≥3 neutropenia 19%, G≤2 fatigue 48%	^262^	2011
	PF‐03814735	1	NCT00424632	Various	Completed	57	PF‐03814735	ORR 0%, DCR 37%	G≥3 neutropenia 21%	^263^	2011
	SNS‐314	1	NCT00519662	Various	Completed	32	SNS‐314	ORR 0%, DCR 19%	No G≥3 > 15%	^264^	2009
	AS703569	1	NCT00391521	Various	Completed	92	AS703569	ORR 0%, DCR 42%	G≥3 neutropenia 17%, all grade fatigue 36%, all grade nausea 37%	^265^	2013
	ENMD‐2076	1	NCT00658671	Various	Completed	67	ENMD‐2076	ORR 3%, DCR 88%	G≥3 37%, hypertension 21%	^266^	2011
		2	NCT01639248	mTNBC	Completed	41	ENMD‐2076	ORR 5%, CBR 17%, mPFS 1.8 mo	G≥3 hypertension 39%, fatigue 10%	^267^	2018
	Ilorasertib (ABT348)	1	NCT01110486	Various	Completed	58	Ilorasertib	ORR 4%	G≥3 all 55%, G≥3 hypertension 17%, all grade fatigue 48%, G≥3 neutropenia 3%	^268^	2018
		1	NCT02540876	Various (CDKN2A‐deficient)	Completed		Ilorasertib				
		2	NCT02478320	Various (CDKN2A‐deficient)	Completed		Ilorasertib				

Abbreviations: CBR, clinical benefit rate; CDK4/6i, CDK4/6 inhibitor; DCR, disease control rate (defined as the sum of complete response, partial response and stable disease as best response); DLT, dose limiting toxicity; G, grade as per CTCAE; HR^+^/HER2^−^, hormone receptor‐positive, HER2‐negative; mBC, metastatic breast cancer; Mo, months; ORR, objective response rate; TNBC, triple negative breast cancer.

To date, only CFI‐4000945 has been tested in clinical trials. Its first‐in‐human trial enrolled 52 patients with advanced solid tumours including four BC patients.^109^ An acceptable safety profile was reported, including fatigue (37%), nausea (21%) and neutropenia (21%), generally of low‐grade. Dose‐dependent neutropenia was observed, of grade ≥3 in 19% at the highest dose level.^109^ Although there was limited anti‐tumour activity in the phase 1 trial that included refractory solid tumours (ORR 2%, CBR 8%), the favourable tolerability profile and strong preclinical rationale led to further therapeutic development in solid tumours across four phase 2 trials, including two BC trials. Preliminary data from a phase 2 study investigating CFI‐400945 monotherapy were recently presented. Among 27 HR^+^/HER2^−^–mBC patients with a median of three prior therapies, including 75% with prior CDK4/6i, three (11%) achieved partial response and seven (26%) continued on treatment beyond 6 months, with a 33% incidence of grade ≥3 neutropenia but otherwise good clinical tolerability.^110^ Among 13 patients in the TNBC cohort, one patient had stable disease for more than 6 months, but no objective responses were observed. These findings are consistent with another phase 2 study that tested CFI‐400945 in combination with the PD‐L1 immune checkpoint inhibitor durvalumab in unselected, pre‐treated TNBC patients. In this trial, two (15%) out of 13 patients had a short‐lived stable disease as best response.^111^ Overall, CFI‐400945 continues its clinical development in HR^+^/HER2^−^–mBC and the results from its correlative program are awaited to identify those patients who can derive the most benefit.

## 4 G2/M PHASE TRANSITION

The initiation of mitosis is ultimately regulated by the activation of the CDK1/cyclin B complex. DNA damage and replication stress checkpoints prevent the accumulation and propagation of genetic errors during cell division. DDR‐related kinases such as ATM and CHK2, and ATR and CHK1, prevent the G2/M transition by activating serine‐threonine kinases WEE1 and PKMYT1, which maintain inhibitory phosphorylation of CDK1 (Figure 1).^112^ Conversely, the degradation of both WEE1 and PKMYT1 is mediated by their phosphorylation by CDK1 and PLK1 in the absence of DNA damage.^113^, ^114^ CDK1/cyclin B complex activation is also critically regulated by aurora kinase A (AURKA), which additionally activates PLK1 to promote mitosis entry.

### 4.1 WEE1

WEE1 and PKMYT1 have been proven essential for cancer cell viability in CRISPR‐Cas9 screens and are consistently found upregulated in solid tumours, where they are thought to maintain tolerable levels of genetic instability.^115^, ^116^ There is, however, scarce data about their role as prognostic biomarkers, with their expression linked to more aggressive phenotypes and worse outcomes in gene datasets of TN and HR^+^ BC.^117^, ^118^, ^119^ The potential of WEE1 as a therapeutic target in BC was substantiated more than a decade ago through RNAi screens of the human kinome.^120^, ^121^ Several WEE1 inhibitors have been developed with the goal of causing mitotic catastrophe by abrogating the G2/M checkpoint, thereby forcing entry into mitosis despite an intolerable burden of genomic damage.

The first compounds, PD0166285 and PD0407824, exhibited modest anti‐proliferative effect and reduced selectivity, and did not reach clinical development.^122^, ^123^ Most of the available preclinical and clinical studies on WEE1 targeting in BC have used adavosertib, a highly selective, ATP‐competitive, small‐molecule inhibitor. In TNBC, synergistic effects have been shown with the combination of adavosertib with platinum chemotherapy and other DDR inhibitors such as olaparib or ATRi in cell line and PDX models.^124^, ^125^, ^126^ Moreover, the higher expression of cyclin E (*CCNE*) has been described as a predictive biomarker for adavosertib monotherapy in TNBC cell lines.^124^ These studies emphasise the potential use of WEE1i to generate homologous recombination deficiency (HRD) and (re)sensitise tumours to agents targeting DNA or its repair machinery. More recent data suggest that WEE1 may also play a prominent role in CDK4/6i‐resistant HR^+^/HER2^−^–mBC. By means of siRNA or adavosertib use, WEE1 abrogation significantly decreased cell proliferation and induced apoptosis and G2/M arrest in several endocrine‐ and CDK4/6i‐resistant cell lines, an effect observed independent of the *RB1* status.^71^, ^118^, ^127^

Four WEE1i, adavosertib, Debio 0123, IMP7068 and ZN‐c3, have entered clinical trials (Figure 2 and Table 2). Adavosertib was first explored in two different phase 1 trials in monotherapy and in combination with gemcitabine, cisplatin or carboplatin.^128^ ^,^ ^129^ In one of them, two out of eight patients carrying *BRCA1/2* mutations, none of them with BC, showed confirmed partial responses. From a total of 25 patients enrolled, common toxicities included myelosuppression (40%) and nausea, vomiting and diarrhoea (60%), mostly of grade ≤2.^128^ In another phase 1 trial, adavosertib was given with standard chemotherapy including gemcitabine or platins.^129^ Of 176 patients evaluable for efficacy, 94 (53%) had stable disease as best response and 17 (10%) achieved a partial response. Further in development, Keenan et al. explored the combination of adavosertib and cisplatin as first‐ or second‐line therapy for mTNBC in a phase 2 trial, where ORR was 26%, CBR was 32% and median PFS was 4.9 months, largely similar to reports of platinum monotherapy in this population in the TNT and TBCRC009 trials.^130^, ^131^ The most common treatment‐related adverse events (TEAEs) were nausea (50%), diarrhoea (35%) and neutropenia (29%), including grade ≥3 diarrhoea (21%) and neutropenia (18%).^132^ This study included an effort to capture correlative biomarkers of response and found that increased post‐treatment CD3/CD4^+^ and CD3/CD8^+^ T‐cell numbers were associated with improved clinical outcome, while no previously reported WEE1i transcriptomic correlates were identified.^132^, ^133^ The strategy of combining WEE1 and PARP inhibition was explored in the phase 2 VIOLETTE trial, which coupled adavosertib and olaparib in mTNBC.^134^ However, this combination was terminated early due to substantial myelotoxicity, and adavosertib development was subsequently discontinued due to its narrow therapeutic window.

While the development of adavosertib has focused principally on ovarian cancer, where it has reached phase 2 testing in combination with chemotherapy, there are other WEE1i with similar preclinical activity and expected superior safety profiles based on ongoing phase 1 trials.^135^ Selective small‐molecule oral inhibitors IMP7068 and ZN‐c3 have recently communicated promising anti‐tumour activity in small dose‐escalating cohorts (CBR 64% with ORR 0% and CBR 44% with ORR 13%, respectively) and a tolerable safety profile (seven grade ≥3 events reported in three out of 24 patients receiving IMP7068).^136^, ^137^, ^138^, ^139^ Similarly, Debio 0123 has been shown tolerable with three grade ≥3 thrombocytopenia events among 38 patients as the most frequent high‐grade TEAE.^140^ These novel inhibitors will eventually inform the potential of WEE1 as a pharmacological target in BC.

### 4.2 PKMYT1

Less studied than WEE1 itself, the related family member PKMYT1 recently emerged as a therapeutic target through a genome‐scale CRISPR–Cas9‐based synthetic lethality screen in cellular models of *CCNE1* amplification.^141^ Following this observation, a selective, orally bioavailable inhibitor was identified, RP‐6306, which has been shown to potently inhibit CDK1/cyclin B activity and produce DNA damage. These findings occurred in a dose‐ and time‐dependent fashion in tumour xenograft models including the implantation of the *CCNE1*‐amplified TNBC cell line HCC1569.^141^ Moreover, the addition of gemcitabine rendered a synergistic effect in the context of DNA replication stress and extended S phase caused by *CCNE1* overexpression.

In an analysis of the kinomes of a series of 20 HR^+^ BC PDX tumours, Chen et al.^119^ recently described PKMYT1 as a marker of hormone independent growth and poor outcome. Of note, *PKMYT1* was found significantly decreased after oestradiol deprivation, an effect likely governed by the oestrogen response elements contained in the regulatory region of the *PKMYT1* gene. These regulatory elements are absent in the *WEE1* gene, thereby suggesting a potential biological rationale for PKMYT1 inhibition over WEE1 in HR^+^ tumours. Altogether, these findings hold promise for further development in BC, particularly where synthetic lethality can be exploited and in luminal BC. RP‐6306 recently entered first‐in‐human clinical studies as monotherapy and in combination with gemcitabine, FOLFIRI and an ATRi, and the gemcitabine combination is also being evaluated in endocrine‐resistant HR^+^/HER2^−^–mBC, with integrated evaluation of putative synthetic lethal biomarkers (Table 2).

### 4.3 Aurora kinases

Once the threshold levels of CDK1 activity are reached, entry into mitosis is triggered by the phosphorylation of a large number of CDK1 substrates, including PLK1, Aurora A (AURKA) and Aurora B (AURKB) mitotic kinases.^142^ Aurora kinases are a family of serine/threonine kinases that play critical functions during mitosis and are consistently overexpressed in malignant tumours. The most studied component is AURKA, which controls the G2/M transition but also exerts pleiotropic functions in centrosome maturation, cytokinesis and the modulation of key transducers of oncogenic signalling.^143^ In contrast, AURKB is essentially involved in chromosome alignment, kinetochore‐microtubule attachment and cytokinesis.

AURKA expression, which is strongly correlated with other markers of proliferation, has been shown to predict worse survival outcomes in both HR^+^/HER2^−^ and TNBC subtypes.^144^, ^145^ AURKA upregulation has been described in endocrine‐resistant HR^+^ models, where its inhibition restored sensitivity to ER blockade and increased the efficacy of ET.^146^, ^147^, ^148^, ^149^ At the genomic level, *AURKA* amplification has been recently reported as a mechanism of resistance to CDK4/6i, and, interestingly, AURKA inhibition has demonstrated a synthetic lethal interaction with *RB1* loss, a well‐known mechanism of acquired resistance to CDK4/6i.^150^, ^151^ In preclinical models of BC, the blockade of AURKA with its ATP‐competitive inhibitor alisertib induced mitotic spindle defects, mitotic delays and apoptosis, and displayed synergistic effects with the addition of paclitaxel.^152^, ^153^, ^154^ Furthermore, our group recently showed the enhanced sensitivity to AURKAi alisertib, barasertib and tozasertib, of a panel of palbociclib‐resistant BC cells, characterised by an increased incidence of micronuclei and segregation errors.^155^

Many drugs targeting aurora kinases have entered clinical development for solid tumours, most of which have been discontinued in early stages due to modest activity, toxicity or to focus in haematological malignancies (Figure 2 and Table 2).^156^ Dose escalation of pan‐aurora kinase inhibitors were limited by frequent toxicities, including neutropenia, fatigue, diarrhoea and hypertension. While selective inhibitors displayed a more favourable safety profile, the lack of anti‐tumour responses as single agents diminished enthusiasm for further development.^157^

Alisertib is the only AURKAi currently in development in BC. Early phase studies evaluating alisertib alone or in combination with fulvestrant in endocrine‐resistant HR^+^/HER2^‐^–mBC demonstrated a favourable safety profile and promising anti‐tumour activity.^158^ Three phase 2 trials have tested alisertib alone or in combination with fulvestrant or paclitaxel in advanced BC.^159^, ^160^ In the first phase 2 study, alisertib monotherapy demonstrated a manageable safety profile in a cohort of 53 BC patients, with an ORR of 18% in HR^+^ or HER2^+^ BC patients resistant to ET, but minimal activity in TNBC.^161^ In the phase 2 study by Haddad et al.,^162^ 91 patients who had previously received ET and CDK4/6i were treated with alisertib alone or in combination with fulvestrant. For alisertib monotherapy, ORR was 20%, 24‐week CBR was 41%, and median PFS was 5.6 months; all similar to the combination where ORR was 20%, CBR was 29% and median PFS was 5.4 months.^162^ The correlative analyses published with this study are based solely on *ER* and *AURKA* expression in pre‐treatment biopsies, where a positive *AURKA* expression was significantly associated with a shorter PFS in the monotherapy arm but not in the combination arm, and with additional studies underway.^162^ Alisertib in combination with paclitaxel has been compared with paclitaxel monotherapy in 139 HR^+^/HER2^−^ endocrine‐resistant patients, only 20% of whom had received prior CDK4/6i. ORR was similar in both treatment arms (31 vs. 34%), while median PFS was significantly longer with the combination (10.2 vs. 7.1 months).^160^ Altogether these trials demonstrate that alisertib is tolerable but associated with an incidence of grade ≥3 neutropenia ranging from 40% in monotherapy to 60% in combination with paclitaxel. Despite the potential interest of further development in the post‐CDK4/6i treatment landscape, no trials in this population are currently registered.

### 4.4 PLK1

Functionally intertwined with AURKA, mainly as a downstream phosphorylation substrate, PLK1 is important in a variety of functions including the regulation of the G2/M checkpoint, spindle formation and chromosome segregation, with emerging evidence also pointing to a role in DDR.^163^, ^164^, ^165^, ^166^ In the cell cycle, PLK1 promotes mitotic entry by upregulating CDK1 and inhibiting WEE1 and PKMYT1 (Figure 1). In BC, PLK1 signalling has been shown to cooperate in ER‐dependent gene transcription, and its genetic or pharmacologic inhibition led to G2/M arrest in TNBC cell lines and to tumour shrinkage in *CCND1*‐driven PDX models of acquired palbociclib resistance.^167^, ^168^, ^169^

A number of PLK1 inhibitors have been identified but most of these molecules displayed limited anti‐tumour activity and poor selectivity in preclinical models.^170^ The clinical development of PLK1 inhibitors, mainly ATP‐competitive volasertib and rigosertib, has focused on haematological malignancies rather than solid tumours, where modest activity has been reported along with considerable myelotoxicity (Table 2).^171^, ^172^, ^173^, ^174^, ^175^, ^176^

## 5 MITOTIC PROGRESSION

### 5.1 TTK

The segregation of sister chromatids during mitosis is under the mechanical control of the mitotic spindle, a highly conserved apparatus nucleated by the centrosomes and composed of microtubules and motor proteins. Once that chromosomal kinetochores are attached to the spindle microtubules and aligned at the metaphase plate, the E3 ubiquitin ligase activity of the anaphase‐promoting complex (APC) governs the progression of anaphase and completion of mitosis.^177^ In the presence of chromatid missegregation, the APC is inhibited by the SAC (Figure 1).^178^ One of the critical regulatory steps is the recruitment of TTK, also known as Monopolar spindle 1 (MPS1), a serine/threonine and tyrosine kinase, to unattached kinetochores, where it activates the SAC to block mitosis.^179^

These observations highlight TTK as an appealing target for drug development, where its inhibition can induce premature anaphase, aneuploidy and intolerable genomic instability. TTK is overexpressed in BC, where it correlates with histologic grade and increased aneuploidy.^180^, ^181^, ^182^ However, its role as a biomarker remains unclear.

TTK inhibition has been shown to reduce proliferation and invasiveness in BC cells lines, and tumour growth in BC xenograft models, showing particular selectivity for highly aneuploid cells.^182^, ^183^, ^184^, ^185^, ^186^ Given the mechanistic rationale of combining TTKi, which override the SAC, with taxane‐based chemotherapy, which engages this checkpoint, substantial preclinical and clinical investigation has focused on such combinations. Several ATP‐competitive TTKi have been studied in preclinical models and a few have reached early phase clinical trials. The first‐generation TTKi SP‐600125 and reversine lacked selectivity and targeted JNK or AURKB, while subsequent agents such as PF‐7006 and PF‐3837 were discontinued due to their limited therapeutic window in preclinical models.^187^, ^188^, ^189^

Clinical data is available from BAY1161909, BAY1217389, CFI‐402257 and S81694, with a particular focus on their combination with paclitaxel (Figure 2 and Table 2). The first clinical evidence of TTK inhibition in patient cohorts was provided by the phase 1 trial evaluating the lead compound BAY1161909 in combination with paclitaxel.^190^ Despite an acceptable safety profile and signs of anti‐tumour activity, with confirmed responses in 14% of patients and DCR ranging from 43 to 60% depending on the dose of paclitaxel (75 vs. 90 mg/m^2^), BAY1161909 was deprioritised in favour of BAY1217389.^190^ Phase 1 data from the combination of the follow‐up compound and paclitaxel in 75 patients, one third of whom had BC, showed concerning bone marrow toxicity, with grade ≥3 neutropenia in 32% of patients and febrile neutropenia in 16% of patients.^191^ Despite an ORR of 32% and DCR of 78%, the myelotoxicity of the combination may limit further development.

Another orally active TTKi, CFI‐402257, has shown preclinical monotherapy and taxane‐combination activity in HR^+^/HER2^−^ and TNBC models.^184^, ^192^, ^193^ Of note, enhanced cytotoxicity has been observed in a subset of CDK4/6i‐resistant models, including those harbouring loss of *RB1*.^155^, ^184^, ^193^ Currently, two phase 1/2 trials are underway evaluating CFI‐402257 in advanced solid tumours and BC. One of them is evaluating CFI‐402257 in monotherapy and in combination with fulvestrant, integrating a dose escalation part in patients with advanced solid tumours and three expansion cohorts at the recommended phase 2 dose (RP2D) including TNBC and HR^+^/HER2^−^–mBC patients progressing on AI and CDK4/6i.^194^ A favourable toxicity profile was reported from 66 patients enrolled across dose escalation and expansion cohorts, 25 of whom had BC, including low‐grade fatigue (47%), nausea (46%) and diarrhoea (32%). Only 9% of patients experienced all‐grade neutropenia, 6% being grade ≥3, the latter occurring in patients allocated to the declared RP2D dose or higher.^194^ The overall 6‐month CBR was 12% and ORR was 5%; however, all the 3 patients with confirmed responses had HR^+^/HER2^−^–mBC, with an average of 7 prior systemic therapies including CDK4/6i. In combination with fulvestrant in 20 HR^+^/HER2^−^ patients, ORR was 10% and 6‐month CBR 25%, with prolonged benefit observed in several patients. In a separate trial testing the combination of CFI‐402257 and paclitaxel, safety and preliminary efficacy results were recently presented from 29 HER2^−^ BC patients, including 90% HR^+^ of whom 75% had previously been treated with CDK4/6i.^195^, ^196^ The ORR was 8%, CBR was 55% and, consistent with the known toxicity profile of paclitaxel, the most frequent clinical adverse events included fatigue (72%), nausea (52%), diarrhoea (45%) and peripheral neuropathy (45%). As expected with weekly paclitaxel, neutropenia was almost universal; grade ≥3 neutropenia was observed in 70% of patients with some dose‐dependency. Following its manageable safety profile and encouraging preliminary efficacy in heavily pre‐treated HR^+^/HER2^−^–mBC, an ongoing study is further evaluating CFI‐402257 in combination with fulvestrant in HR^+^/HER2^−^–mBC after disease progression to ET plus CDK4/6i (TWT‐203).

As CFI‐402257 progresses through clinical trials, correlative analyses will permit the characterisation of biomarkers and guide patient stratification. Our group has identified a two‐gene expression signature within the APC components (*ANAPC4* and *CDC20*) that is strongly correlated with CFI‐402257 activity in TNBC cell lines.^184^ Moreover, we have observed an increased cytotoxicity in CDK4/6i‐resistant models harbouring *RB1* loss.^155^, ^184^, ^193^ In light of this body of preclinical and clinical knowledge, CFI‐402257 was recently granted US FDA Fast Track Designation for further development both as monotherapy and in combination with fulvestrant for HR^+^/HER2^−^–mBC patients who have progressed on CDK4/6i.

Developed in parallel, Schöffski et al.^197^ recently communicated results on the safety and preliminary anti‐tumour activity of the first‐in‐human study of the intravenous TTKi S81694 in patients with solid tumours. Among 38 participants, the most common adverse events included fatigue (58%), anaemia (45%) and nausea (32%), with mild haematological toxicity including grade ≥3 neutropenia in 11% of patients.^197^ Among 35 efficacy‐evaluable patients, ORR was 6% and DCR was 43%. RP2D, however, was not defined due to the sponsor's decision to stop enrolment to monotherapy and prioritise S81694 testing in combination with cytotoxic agents.

### 5.2 KIF18A

A mitotic kinesin motor protein that localises to the plus‐end of kinetochore microtubule spindle fibres, KIF18A plays an essential role in cell division in aneuploid cancer cells with high chromosomal instability.^198^, ^199^, ^200^ In BC, *KIF18A* expression levels are increased and associated with higher tumour grade, metastases and shorter survival.^201^ Genetic ablation of *KIF18A* led to proliferation inhibition via centrosome fragmentation and mitotic arrest both in vitro and in vivo, primarily affecting tumour cells with chromosome instability while inducing relatively low toxicity in diploid cells.^198^, ^199^, ^201^ These findings, combined with promising preclinical results in other tumour types, have paved the way for the development of pharmacological inhibitors such as sovilnesib and VLS‐1488, currently undergoing phase 1 testing with no clinical data yet reported.^200^, ^202^

## 6 PERSPECTIVE OF CELL CYCLE INHIBITORS IN THE CURRENT THERAPEUTIC LANDSCAPE

**FIGURE 3:**
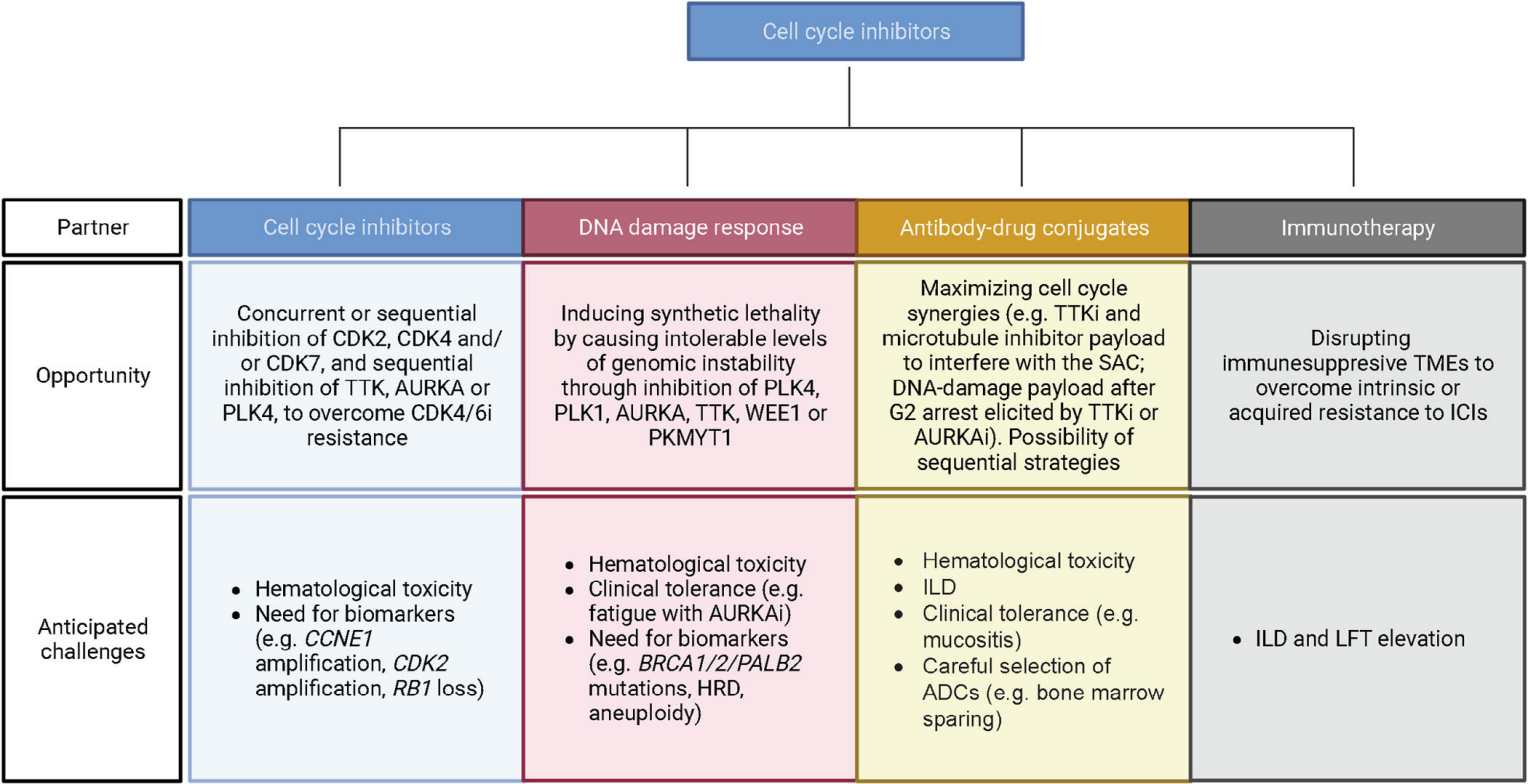
Rationale and challenges for the potential development of cell cycle inhibitors in combination with other therapies in breast cancer. ADC, antibody–drug conjugate; HRD, homologous recombination deficiency; ICI, immune checkpoint inhibitor; ILD, interstitial lung disease; LFT, liver function tests; TME, tumour microenvironment.

Given the rapid introduction of novel endocrine agents and antibody–drug conjugates (ADCs) into the therapeutic landscape, the successful development of novel agents targeting the cell cycle will likely require robust anti‐tumour activity, manageable toxicity and, in contrast to the initial use of CDK4/6i in combination with ET, biomarker enrichment. We envision four areas of strategic interest (Figure 3).

### 6.1 Targeting of cell cycle components either concurrently or upon resistance to CDK4/6i in HR^+^/HER2^−^–mBC

Important advances in the treatment of HR^+^/HER2^−^–mBC have recently focused on targeting *ESR1* mutations, where oral SERDs have demonstrated activity and ctDNA‐guided switching of the endocrine partner contributes to disease control in the face of emerging AI resistance.^203^, ^204^ While *ESR1* mutations are the best characterised drivers of acquired resistance to ET and CDK4/6i combinations, CDK4/6i activity does not seem to be influenced by them.^205^, ^206^, ^207^ Most genomic alterations described in CDK4/6i‐resistant tumours converge on their ability to override the G1/S checkpoint. Blocking CDK2 or CDK7, known to underlie CDK4/6i resistance, have attracted attention as potential strategies to restore sensitivity. In this regard, switching CDK4/6i or switching to other CDKi such as the CDK2/4/6i ebvaciclib and CDK7i samuraciclib could have a role, and have shown some activity in the post‐CDK4/6i setting; however, much remains to be learned about the predictors of efficacy for these strategies, the optimal agents and endocrine combinations, and how they might compare. While samuraciclib is currently being tested in combination with newer endocrine agents in the post‐CDK4/6i setting, efforts to elucidate the benefit of targeting CDK2 and CDK4 separately or together post‐CDK4/6i, CDK2i combined with CDK4/6i, or CDK4i in treatment‐naive patients are underway (Table 1). These trials will provide valuable data not only to understand CDK2, CDK4 and CDK7 targeting but also the optimal treatment sequencing or correlatives of benefit.

Encouraging preclinical evidence and manageable toxicity profiles in phase 1 trials support the development of PLK4i, AURKAi and TTKi in CDK4/6i‐resistant HR^+^/HER2^—^–mBC. Notably, TTKi CFI‐402257 has shown enhanced preclinical activity in models harbouring *RB1* loss, a key mechanism of CDK4/6i resistance, and both TTKi and AURKAi seem to be tolerable in combination with fulvestrant and paclitaxel. Due to overlapping myelosuppression, the development of combinations of these compounds with CDK4/6i in the front‐line setting is unlikely; however, an understanding of the clinical activity in the face of emerging resistance could support innovative approaches to develop sequential strategies. In the light of upfront ADC use post‐CDK4/6i, PLK4i, AURKAi or TTKi are most likely to thrive in combination with SERDs and novel ETs as chemo‐sparing regimens in molecularly selected populations, or in monotherapy as a later line of therapy.

### 6.2 Inducing synthetic lethality in combination with DDR agents such as PARPi or ATRi in TNBC or HRD disease

PARPi olaparib and talazoparib are the only DDR agents approved for HER2^−^–mBC patients with *gBRCA1/2* mutations. Many others are currently in development with the goals of overcoming PARPi resistance, expanding efficacy against tumours with somatic DDR mutations and HRD and reducing toxicity.^208^, ^209^ The interplay of DDR and cell cycle vulnerabilities provides an appealing therapeutic opportunity. Most TNBC harbour DDR and p53 defects that result in an inactive G1/S checkpoint, thus increasing the relevance of the G2/M checkpoint to respond to DNA damage.^126^ Moreover, an association between *gBRCA1/2* and early resistance to CDK4/6i in luminal tumours is being increasingly recognised, where HRD genomic features may also be enriched after progression to CDK4/6i.^210^, ^211^, ^212^, ^213^, ^214^, ^215^ Increasing replication stress and genomic instability by interfering with cell cycle checkpoints may thus be leveraged to generate synthetic lethality or to re‐sensitise tumours to DDR‐based approaches.

Synergistic efficacy of CDK4/6i and PARPi has been observed in both *RB1*‐proficient and ‐deficient BC cell lines, which is notable given CDK4/6i causes G1/S arrest in *RB1*‐proficent cells and prevent S phase entry, where PARPi exerts their cytotoxicity.^216^, ^217^, ^218^ This approach is currently being explored in the HOPE study, which combines olaparib, palbociclib and fulvestrant in *BRCA1/2*‐mutant HR^+^/HER2^−^–mBC patients. Despite the preclinical and clinical rationale, a substantial burden of haematological toxicity is likely. In small studies, response rates of 10−25% have been reported with WEE1i adavosertib or unselective CDKi selicilib in patients carrying g*BRCA1/2* mutations.^65^, ^128^ However, combinatorial strategies have been challenged by considerable haematological toxicity, with some examples including the combination of adavosertib with PARPi olaparib, or the combination of adavosertib, PLK1i volasertib and unselective CDKi with platins.^62^, ^132^, ^172^, ^219^

Opportunities may emerge with more tolerable DDR agents such as PARP1i and ATRi. The synthetic lethality of PARPi largely relies on PARP1 inhibition, with PARP2 being essentially involved in haematological homeostasis. Thus, novel PARP1‐selective inhibitors such as AZD5305 or AZD9574 that are associated with less myelotoxocity may be more favourable partners for combination testing with cell cycle inhibitors. ATRi such as berzosertib or ceralasertib have also shown a milder safety profile amenable for combination.^219^, ^220^, ^221^, ^222^ Since WEE1 and PKMYT1 are direct downstream transducers of ATR‐mediated DDR, inhibitors of these molecules are well positioned for a cooperative effect. In this regard, the combination of adavosertib with ceralasertib or with an inhibitor of the anti‐apoptotic protein BCL‐XL led to synergistic anti‐tumour effects with a significant therapeutic window in vivo and in a TNBC cell model, respectively.^223^, ^224^ Furthermore, the genetic abrogation of *PKMYT1* has been shown to restore sensitivity to adavosertib in vitro, and a phase 1 clinical trial investigating the combination of the PKMYT1i RP‐6306 with ATRi RP‐3500 is underway.^225^

### 6.3 Combination of ADCs and cell cycle inhibitors

While additive or synergistic effects are plausible, preclinical evidence testing this strategy is lacking that could shed light into cell cycle synergies or enhanced genomic instability. The improved therapeutic index of ADCs over standard chemotherapies and their activity on selective tumour populations make them suitable partners for targeted agents, thus opening several therapeutic opportunities. First, the combination of cell cycle inhibitors with chemotherapies is feasible as demonstrated by the tolerability of the combination of paclitaxel with TTKi CFI‐402257 and BAY1217389, and with AURKAi alisertib.^160^, ^191^, ^195^ One can expect a higher efficacy and less toxicity from the combination of TTKi or AURKAi and microtubule‐targeting ADCs in development. Second, ADCs carrying DNA‐damaging agents that act on the S‐phase and lead to G2 arrest (e.g. topoisomerase inhibitors, anti‐metabolites such as gemcitabine) could be suitably coupled with targeted inhibition of G2/M, as a way to render cytotoxicity by producing catastrophic genomic aberrations. Third, the sequential use of ADCs and cell cycle inhibitors, where ADCs may eradicate resistant clones and cell cycle inhibitors provide maintenance therapy thereafter. Modern ADCs with stable linkers, such as trastuzumab deruxtecan and datopotamab deruxtecan that cause substantially less myelosuppression than their counterparts may well provide exploratory candidates, although we expect this list to grow with the progressive optimisation of ADC properties and molecular selection of target populations. Finally, for the potent cell cycle inhibitors that have limited therapeutic index, the possibility of formulating these as ADCs may enable the development of molecularly‐guided payloads that exploit cell cycle anomalies and overcome resistance to known agents.

### 6.4 Leveraging an increased tumour immunogenicity in combination with immunotherapeutics

Mounting preclinical evidence indicates that CDK4/6i sensitise in vitro and in vivo models to anti‐PD‐1 blockade, mainly via stimulation of tumour cell antigen presentation, inhibition of the proliferative capacity of T regulatory cells and increase of T cell inflammatory signatures.^226^, ^227^, ^228^ The modest efficacy of anti‐PD‐1 therapy in the presumably “cold” HR^+^/HER2^−^ breast tumours, the niche of CDK4/6i, provides an opportunity to investigate the immune‐priming effects of cell cycle inhibition as well as the features associated with acquired resistance that could enhance sensitivity to such strategies. In the study conducted by Yuan et al.,^229^ 23 HR^+^/HER2^−^–mBC patients received palbociclib, pembrolizumab and letrozole, achieving an ORR of 55% that included five complete responses out of 16 patients treated as first‐line. Moreover, intriguing results were observed in the PACE study, a randomised phase II study where the switch of AI to fulvestrant after progression to AI and CDK4/6i did not improve PFS, but where addition of avelumab to fulvestrant and CDK4/6i nearly doubled ORR and PFS.^49^ The combination of abemaciclib plus pembrolizumab has also demonstrated anti‐tumour activity, but caused high rates of interstitial lung disease and severe transaminase elevations which precluded further development, and similarly the phase Ib trial studying the combination of ribociclib and anti‐PD‐1 spartalizumab has been terminated due to undisclosed safety implications.^230^, ^231^

Preclinical evidence underscores the interest of blocking other cell cycle components to sensitise tumours to immune‐checkpoint inhibition. PLK1i volasertib and WEE1i adavosertib have been shown to enhance response to anti‐PD‐1 agents in models of lung cancer by increasing PD‐L1 expression, activating interferon pathways and stimulating cytotoxic T cell infiltration, and our group observed a similar outcome with TTKi CFI‐402257 in a murine model of colon cancer.^192^, ^232^, ^233^ Furthermore, CFI‐402257 was recently found to restore anti‐PD‐1 efficacy in a model of *KRAS‐LKB1*‐mutant lung cancer, which are intrinsically resistant to PD‐1 blockade via epigenetic abrogation of STING.^234^ There are, however, limited clinical data testing this strategy, with the combination of the unselective CDKi dinaciclib and pembrolizumab, and of CFI‐400945 and durvalumab, being safe but exhibiting limited activity in unselected patients with heavily pre‐treated mTNBC.^69^, ^111^

## 7 CONCLUSION

The deregulation of the cell cycle enables BC cells to proliferate and thrive despite adverse environments and cumulative genomic instability. Both ER‐dependent and ‐independent mechanisms modulate cell cycle checkpoints, where CDK4/6 inhibition has attained remarkable success in HR^+^ tumours. Despite its appeal as a pharmacological target, the complexity of the cell cycle, with pleiotropic regulatory pathways and functional redundancy of its components, has hindered therapeutic development. Our growing understanding of the science underlying cell cycle deregulation along with advances in drug design provide an expanded therapeutic window for clinical development. The successful incorporation of CDK4/6i in metastatic and now early HR^+^/HER2^−^–BC has created the new entity of CDK4/6i‐resistant disease, with unique treatment‐resistant genomic alterations. Bolstered by strong preclinical evidence and careful consideration of molecular determinants of sensitivity, novel inhibitors may be well‐positioned to overcome CDK4/6i resistance, induce synthetic lethality in genomically unstable tumours and boost immunogenicity and sensitivity to immunotherapeutics, either as monotherapy or combined with CDK4/6i, DDR agents or immune checkpoint inhibitors, respectively. However, the therapeutic landscape is rapidly expanding with the introduction of novel endocrine agents and ADCs post‐CDK4/6i for HR^+^/HER2^−^ disease, and of immune‐chemotherapy combinations and ADCs in TNBC. Demonstrating the clinical benefit of these novel cell cycle inhibitors will require rational, science‐driven clinical trial designs and comprehensive efforts to identify predictive biomarkers.

## AUTHOR CONTRIBUTION

JFA, PLB and DWC contributed to the conception and scope of the study. JFA wrote the first draft and composed the figures/tables. All authors critically reviewed the manuscript and approved the submitted version.

## CONFLICT OF INTEREST STATEMENT

D. W. C.: Consulting or Advisory Role—AstraZeneca; Daiichi Sankyo; Eisai; Gilead Sciences; GlaxoSmithKline; Inflex; Inivata/NeoGenomics; Lilly; Merck; Novartis; Pfizer; Roche/Genentech and Saga. Research Funding—AstraZeneca (Inst); GlaxoSmithKline (Inst); Guardant Health (Inst); Inivata/NeoGenomics (Inst); Knight Therapeutics (Inst); Merck (Inst); Pfizer (Inst); ProteinQure (Inst); and Roche/Genentech (Inst). Patents, Royalties, Other Intellectual Property—Patent (US62/675,228) for methods of treating cancers characterized by a high expression level of spindle and kinetochore associated complex subunit 3 (ska3) gene. P. L. B.: Consulting or Advisory Role—Seattle Genetics; Lilly; Amgen; Merck; Gilead Sciences; Zymeworks; Repare Therapeutics; BMS; Pfizer. Research Funding—Bristol‐Myers‐Squibb (Inst); Sanofi (Inst); AstraZeneca (Inst); Genentech/Roche (Inst); GlaxoSmithKline (Inst); Novartis (Inst); Merck (Inst); Seattle Genetics (Inst); Amgen (Inst); Bicara (Inst); Zymeworks (Int); Medicenna (Inst); Bayer (Inst); Takeda (Inst). J. F. A. declares no competing interests. CFI‐402257 and CFI‐400945 were developed at the University Health Network (the authors' institution).

## ETHICS STATEMENT

Not applicable.

## Supporting information

Supporting Information

## Data Availability

Not applicable.
