# Supplementary material for: Seize the engine: Emerging cell cycle targets in breast cancer
Source: Clin Transl Med. 2024 Jan 24;14(1):e1544. doi: 10.1002/ctm2.1544 (PMC10807317; doi:10.1002/ctm2.1544)
Supplement: Supplementary file 1 — Supporting Information [file CTM2-14-e1544-s001.docx]

**Supplemental information**

**Title**

Seize the engine: emerging cell cycle targets in breast cancer

**Running title:**

Targeting the cell cycle in breast cancer

**Authors**

Jesús Fuentes-Antrás^1,2^, Philippe L Bedard^1^, and David W Cescon^1†^

**Affiliations**

^1^Division of Medical Oncology and Hematology, Department of Medicine, Princess Margaret Cancer Centre, University Health Network, University of Toronto, Toronto, Ontario, Canada

^2^ New Experimental Therapeutics (NEXT) Oncology, Hospital Universitario QuironSalud Madrid, Madrid, Spain

^†^Corresponding author. Email: [dave.cescon@uhn.ca](mailto:dave.cescon@uhn.ca)

700 University Avenue, Suite 7-624, Toronto, Ontario, M5G1Z5 Canada

t. (416) 946 4501 ext. 2245; f. (416) 946-6546, e. [dave.cescon@uhn.ca](mailto:Lillian.siu@uhn.ca)

**Table s1. Drug development targeting cyclin-dependent kinases 4 and 6 (CDK4/6s).** Active, completed, and terminated trials focused on breast cancer are shown.

| **Drug** | **Stage** | **NCT** | **Patient Cohort** | **Status** | **Intervention** | **Year first posted** |
| --- | --- | --- | --- | --- | --- | --- |
| Palbociclib  (PD-0332991) | FDA/EMA approved | 249 studies | See dedicated reviews such as ^38,40^ | | | |
| Ribociclib (LEE011) | FDA/EMA approved | 107 studies |  |  |  |  |
| Abemaciclib (LY2835219) | FDA/EMA approved | 109 studies |  |  |  |  |
| Trilaciclib (G1T28) | 2 | NCT02978716 | mTNBC | Terminated (per protocol, not stopped due to safety concerns) | + carboplatin and gemcitabine | 2016 |
|  | 2 | NCT01042379 | BC early stage | Active, not recruiting | + (neo)adjuvant chemotherapy +/- trastuzumab and pertuzumab | 2020 |
|  | 2 | NCT05112536 | TNBC early stage | Completed | + (neo) adjuvant chemotherapy | 2021 |
|  | 3 | NCT04799249 | mTNBC | Active, not recruiting | + carboplatin and gemcitabine | 2021 |
|  | 2 | NCT05113966 | mTNBC | Active, not recruiting | + sacituzumab govitecan | 2021 |
| Lerociclib (G1T38) | 1/2 | NCT02983071 | HR^+^/HER2^-^ | Active, not recruiting | + ET | 2016 |
|  | 2 | NCT05085002 | HR^+^/HER2^-^ | Active, not recruiting | + ET | 2021 |
| Dalpicilib (SHR6390) | 1/2 | NCT03772353 | HR^+^/HER2^+^  (CDK4/6i-naïve) | Active, not recruiting | + ET and pyrotonib | 2018 |
|  | 2 | NCT04997798 | HR^+^/HER2^+^  early stage | Active, recruiting | + ET and trastuzumab and pyrotinib | 2021 |
|  | 2 | NCT05640778 | HR^+^/HER2^-^  early stage | Active, recruiting | + neoadjuvant ET | 2022 |
|  | 1/2 | NCT05574881 | HR^+^/HER2^+^  (CDK4/6i-naïve) | Active, not recruiting | + ET and trastuzumab and pertuzumab | 2022 |
|  | 2 | NCT05638594 | HR^+^/HER2^+^  early stage | Active, recruiting | + ET and trastuzumab and pyrotinib | 2022 |
|  | 2 | NCT05176080 | HR^+^/HER2^+^  early stage | Active, not recruiting | + ET and trastuzumab and pyrotinib | 2022 |
|  | 2 | NCT06107673 | HR^+^/HER2^+^  early stage | Active, recruiting | + neoadjuvant ET | 2023 |
|  | 3 | NCT05861830 | HR+/HER2-  (post-CDK4/6i) | Active, recruiting | + ET | 2023 |
|  | 2 | NCT06133088 | HR^+^/HER2^-^  (post-CDK4/6i) | Active, recruiting | + ET | 2023 |
|  | 2 | NCT05800756 | HR^+^/HER2^+^ | Active, recruiting | + ET and pyrotonib and trastuzumab | 2023 |
|  | 2 | NCT05328440 | HR^+^/HER2^+^  (CDK4/6i-naïve) | Active, recruiting | + pyrotinib | 2023 |
|  | 2 | NCT05759572 | HR^+^/HER2^-^ | Active, recruiting | + ET and apatinib | 2023 |
|  | 2 | NCT05759546 | HR^+^/HER2^-^ | Active, recruiting | + ET and PARPi | 2023 |
| FCN-437c | 3 | NCT05439499 | HR^+^/HER2^-^ (CDK4/6i-naïve) | Recruiting | + ET | 2022 |
|  | 3 | NCT05438810 | HR^+^/HER2^-^ (CDK4/6i-naïve) | Recruiting | + ET | 2022 |
| BPI-16350 | 3 | NCT05433480 | HR^+^/HER2^-^ (CDK4/6i-naïve) | Active, not recruiting | + ET | 2022 |
| Birociclib  (XZP-3287) | 3 | NCT05077449 | HR^+^/HER2^-^ (CDK4/6i-naïve) | Active, recruiting | + ET | 2021 |
| HS-10342 | 2 | NCT05044988 | HR^+^/HER2^-^ (CDK4/6i-naïve) | Active, not recruiting | Monotherapy | 2021 |
| CS3002 | 1 | NCT04162301 | Various tumors | Terminated (business decision) | Monotherapy | 2019 |

**Abbreviations**: CDK4/6i, CDK4/6 inhibitor; FDA, Food and Drug Administration; EMA, European Medicines Agency; ET, endocrine therapy; HR^+^/HER2^-^, hormone receptor-positive, HER2-negative; HR^+^/HER2^+^, hormone receptor-positive, HER2-positive; mBC, metastatic breast cancer; TNBC, triple negative breast cancer.
